# Extubation outcomes in critically ill post-craniotomy patients: A retrospective cohort study

**DOI:** 10.1371/journal.pone.0333732

**Published:** 2025-10-03

**Authors:** Jianfang Zhou, Xu-Ying Luo, Guangzhi Shi, Hong-Liang Li, Guang-Qiang Chen

**Affiliations:** 1 Department of Critical Care Medicine, Beijing Tiantan Hospital, Capital Medical University, Beijing, China; 2 Department of Critical Care Medicine, Beijing Shijitan Hospital, Capital Medical University, Beijing, China; Universitatsklinikum Regensburg, GERMANY

## Abstract

**Objective:**

Extubation failure is common in critically ill post-craniotomy patients. This study aimed to assess extubation outcomes and examine demographic and surgical predictors of extubation success.

**Methods:**

In this retrospective cohort study, we analyzed adult ICU patients from January 2017 to December 2020 with ≥24 hours of both ICU stay and endotracheal intubation. We collected demographic characteristics, surgical parameters, severity scores (APACHE II, SOFA, GCS), and extubation outcomes. Independent predictors of successful extubation were determined through multivariate logistic regression.

**Results:**

Among 1,683 enrolled patients, first-attempt extubation success was achieved in 70.7% (1,190/1,683), declining to 46.6% (135/290) and 28.6% (12/42) for second and third attempts respectively. Subsequent tracheostomy was performed in 19, 3, and 1 patients following first, second, and third successful extubations. At discharge, 1,244 patients (73.9%) maintained extubation success while 348 (20.7%) required tracheostomy. Successful first extubation correlated with significantly reduced ICU stay (median 4 vs 15 days, p < 0.001) and total hospitalization duration (19 vs 30 days, p < 0.001), though mortality rates showed no significant difference (6.0% vs 8.1%, p = 0.106). Multivariable analysis demonstrated that male sex (OR 0.680, 95% CI 0.531–0.871), history of hypertension (OR 0.637, 95% CI 0.489–0.829), supratentorial lesions (OR 1.315, 95% CI 1.016–1.701), SOFA score (OR 0.844, 95% CI 0.791–0.900), and APACHE II score (OR 0.930, 95% CI 0.902–0.958) were independent factors influencing initial extubation success.

**Conclusion:**

Although first-attempt extubation success remains suboptimal in post-craniotomy critical care, nearly half of initial failures achieve success on subsequent attempts. Multivariable analysis identified female sex, absence of hypertension, infratentorial lesions, lower SOFA, and APACHE II score were independent predictors of initial extubation success.

## 1. Introduction

Extubation decision-making in critically ill post-craniotomy patients remains clinically challenging. Premature extubation may result in failure, potentially leading to airway complications, increased nosocomial pneumonia risk, prolonged hospitalization, worsened neurological outcomes, and elevated mortality rates [1]. Conversely, delayed extubation is associated with heightened analgesic/sedative needs, greater incidence of ventilator-associated pneumonia, extended intensive care unit (ICU) and hospital length of stays (LOS), increased healthcare costs [2], and potentially higher mortality [3, 4].

Patients with acute brain injury (ABI) exhibit significantly lower rates of cardiopulmonary dysfunction yet demonstrate higher extubation failure rate when compared to non-neurologic patients [5]. Traditional extubation criteria used in general ICUs, such as rapid shallow breathing index [6], negative inspiratory pressure, and minute ventilation [7, 8], demonstrate limited predictive value for ABI populations, which might be due to fundamental physiological differences. ABI patients often maintain adequate pulmonary mechanics but fail extubation from impaired central respiratory drive, delayed neurological deterioration, or reduced airway clearance capacity, despite meeting conventional extubation criteria [9]. Existing research has established significant associations between extubation outcomes and several clinical factors, including Glasgow Coma Scale (GCS) scores [6], cough reflex [6, 10], and swallowing function [10–15]. However, some patients with preserved consciousness and intact airway reflexes may still fail extubation. This suggests that beyond known factors, demographic characteristics and surgical variables, such as underlying comorbidities, disease severity, surgical site, and etiology of brain injury, may influence extubation outcomes.

The primary study aim was to assess extubation outcomes in this population, with secondary objectives examining the impact of demographic characteristics, disease severity, and surgical factors on extubation success.

## 2. Methods

### 2.1. Study design

This study was a secondary analysis of a retrospective cohort study conducted in the ICU (70 beds) of an academic tertiary hospital. The study protocol received approval from the Institutional Review Board. Prior to patient enrollment, verbal consent was obtained via telephone from either the patients themselves or their next of kin, with written informed consent waived due to the retrospective nature of the research.

We enrolled patients admitted to the ICU between January 2017 and December 2020 who met all inclusion criteria: (1) aged 18 years or older; (2) having undergone craniotomy; (3) requiring ICU admission for ≥24 hours; and (4) receiving endotracheal intubation for ≥24 hours. Exclusion criteria were: (1) patients without intubation weaning or tracheostomy attempts during hospitalization; (2) pregnant or lactating women; and (3) participants in other clinical studies. Between September 15, 2024, and October 8, 2024, we contacted the patients or their next of kin by phone to obtain their verbal informed consent; patients who were unreachable or declined consent were also excluded from the study.

### 2.2. Data collection

Data were collected from December 25, 2024 to February 28, 2025 using standardized case report forms, with patient identification limited to hospital admission numbers. Personally identifiable information (PII) such as names and ID numbers was not collected. All data-related documents were stored in dedicated filing cabinets, and analysis was performed on designated computers to ensure protection against patient information breaches.

To ensure rigorous data quality control, dual independent data entry was performed by two intensive care specialists, with subsequent validation of data integrity and consistency conducted by a senior attending physician. Any discrepancies were adjudicated through consensus discussions with the primary data collectors followed by appropriate revisions. The collected dataset encompassed: (1) baseline demographic parameters; (2) principal neurological diagnoses and pre-existing comorbidities; (3) detailed operative characteristics (including procedural duration and anatomical location); (4) postoperative adverse events (encompassing infectious complications, sepsis, and acute kidney injury); and (5) critical illness severity indices recorded on ICU admission day – specifically, GCS (with the verbal component defaulted to 1 for mechanically ventilated patients) [16], Acute Physiology and Chronic Health Evaluation (APACHE) II scores [17], and Sequential Organ Failure Assessment (SOFA) scores [18].

The data collected in this study, including age, gender, comorbidities, smoking/alcohol status, were all retrievable from standardized medical records. Details of endotracheal intubation or tracheostomy were verifiable through corresponding operation or procedure records. Reasons for reintubation were documented in the structured intubation records. Surgical indications, sites, and durations were recorded in both surgical and anesthesia records. Patients’ discharge status (including mortality) was obtainable from discharge or death records. In our center, APACHE II scores are routinely documented using electronic forms in the medical record system 24 hours after ICU admission, and the SOFA score is assessed once daily, while the GCS is assessed every 2 hours. Given this exceptional data accessibility, missing data was unlikely to occur.

The study institution implemented a standardized extubation protocol mandating daily sedation interruption with exclusion of clinically unstable patients including those demonstrating severe intracranial hypertension or persistent acute respiratory distress syndrome (ARDS). Eligible patients demonstrating hemodynamic/metabolic stability, adequate oxygenation (arterial oxygen partial pressure to inspired oxygen fraction ratio [P_a_O₂/FiO₂] ≥150 mmHg), acceptable ventilation parameters, and minimal secretions underwent spontaneous breathing trials (SBTs) [19]. SBTs were performed via either T-piece or pressure support ventilation (PSV) mode. T-piece trials maintained FiO₂ ≤ 40%, while PSV trials employed ≤8 cmH₂O pressure support, ≤ 8 cmH₂O PEEP, and FiO₂ ≤ 40%. Trial duration was titrated between 30–120 minutes according to individual patient status. Prespecified SBT failure criteria comprised: (1) tachypnea (respiratory rate >35/min) and/or accessory respiratory muscle recruitment; (2) hypoxemia (peripheral oxygen saturation [SpO₂] <90% at FiO₂ > 50%) concurrent with hemodynamic compromise (heart rate >140 beats/minute, > 20% increase from baseline, significant arrhythmias, or systolic blood pressure <90 mmHg); (3) clinically evident distress (agitation, diaphoresis, or marked anxiety).

For patients who successfully completed SBTs but exhibited risk factors for post-extubation stridor, including difficult intubation, prolonged intubation (>6 days), use of larger-diameter endotracheal tubes, female sex, or history of unplanned extubation requiring reintubation [20–24], we performed quantitative cuff leak testing [25, 26]. Patients with an absolute cuff leak volume <110 mL would receive intravenous corticosteroids with subsequent extubation attempted after a 4-hour observation period. The primary medical team determined SBT eligibility, interpreted results, and made final extubation decisions, while certified respiratory therapists performed the actual extubation procedure. Reintubation decisions were made by attending intensivists according to strict institutional criteria: (1) refractory hypoxemia requiring FiO₂ > 0.5 to maintain SpO₂ > 90%; (2) tachypnea >35 breaths/minute with accessory muscle recruitment; (3) cardiopulmonary arrest; (4) impaired cough reflex with inability to clear secretions; (5) acute hypercapnic respiratory failure (P_a_CO₂ > 50 mmHg with arterial pH < 7.35); (6) clinically significant neurological decline; (7) hemodynamic compromise (sustained tachycardia >140 beats/minute, > 20% increase from baseline heart rate, new-onset arrhythmias, or systolic hypotension <90 mmHg); or (8) severe agitation with signs of respiratory muscle fatigue.

We recorded extubation outcomes including: (1) initial extubation results, (2) airway status at discharge (no artificial airway, retained endotracheal tube, or tracheostomy). Extubation success was defined as no reintubation or tracheostomy required within 72 hours post-extubation. Early tracheostomy was defined as performed within 7 days post-intubation, while late tracheostomy was defined as performed beyond 7 days. Patients meeting extubation success criteria might undergo reintubation or tracheostomy after 72 hours; these patients were excluded from subsequent extubation outcome assessments and reintubation cause analysis, while their final discharge airway status was recorded as the actual status at discharge. Secondary outcome measures included hospitalization LOS, ICU LOS, hospitalization costs, in-hospital mortality, and discharge Glasgow Outcome Scale (GOS).

### 2.3. Statistical methods

Categorical variables were summarized as frequency (%) and analyzed using Pearson’s χ² test or Fisher’s exact test, as appropriate. Continuous variables were reported as either mean ± standard deviation for normally distributed data or median with interquartile range (IQR) for non-normally distributed data, with between-group comparisons performed using Student’s t-test (two groups), Mann-Whitney U test (two groups), or Kruskal-Wallis test (three or more groups), followed by Bonferroni-adjusted post hoc comparisons for multiple testing. Time-to-event data for extubation were analyzed using Kaplan-Meier survival curves with between-group differences assessed by log-rank test. Determinants of successful first extubation attempt were evaluated through binary logistic regression modeling, with the outcome variable dichotomized into success versus failure (incorporating both initial extubation failures and patients requiring direct tracheostomy). The multivariate model included all variables demonstrating marginal association (P < 0.20) in univariate screening and clinical significance. All statistical tests were two-sided, with P < 0.05 defining statistical significance.

## 3. Results

### 3.1. Baseline variables

The cohort comprised 1,683 consecutive patients (Table 1), with a median age of 50 years (IQR: 38–61) and male predominance (884/1,683, 52.5%). Hypertension was the most frequent comorbidity (n = 419, 24.9% [95% CI 22.9–27.0]), followed by diabetes mellitus (n = 155, 9.2% [95% CI 7.9–10.7]), pre-existing cerebrovascular disease (n = 87, 5.2% [95% CI 4.2–6.4]), and malignancies (n = 79, 4.7% [95% CI 3.7–5.8]).

**Table 1:** Demographic and outcome comparisons among direct tracheostomy patients and those with first extubation success or failure.

Variables	Total (n = 1683)	Success (n = 1190)	Failed (n = 290)	Tracheostomy (n = 230)	p value
Age (year)^†^	50(38, 59)	48(36, 57)	54(44, 63)	53(40, 60)	<0.001
Male^‡^	884(52.5%)	583(49.0%)	183(63.1%)	118(51.3%)	<0.001
Smoking^‡^	256(15.2%)	162(13.6%)	58(20.0%)	36(15.7%)	0.014
Alcoholism^‡^	129(7.7%)	80(6.7%)	31(10.7%)	18(7.8%)	0.059
Comorbidities					
Hypertension^‡^	419(24.9%)	241(20.3%)	117(40.3%)	61(26.5%)	<0.001
Diabetes^‡^	155(9.2%)	93(7.8%)	32(11.0%)	30(13.0%)	0.003
Tumor^‡^	79(4.7%)	50(4.2%)	22(7.6%)	7(3.0%)	0.034
Cerebrovascular disease^‡^	87(5.2%)	57(4.8%)	17(5.9%)	13(5.7%)	0.531
Coronary heart disease^‡^	50(3.0%)	30(2.5%)	12(4.1%)	8(3.5%)	0.238
Chronic lung disease^‡^	9(0.5%)	6(0.5%)	2(0.7%)	1(0.4%)	0.929
Pneumonia^‡^	540(32.1%)	214(18.0%)	190(65.5%)	136(59.1%)	<0.001
CNS infection^‡^	387(23.0%)	257(21.6%)	78(26. 9%)	52(22.6%)	0.101
Surgical site infection^‡^	39(2.3%)	19(1.6%)	10(3.4%)	10(4.3%)	0.005
Sepsis^‡^	391(23.2%)	151(12.7%)	142(49.0%)	98(42.6%)	<0.001
AKI^‡^	187(11.1%)	96(8.1%)	47(16.2%)	44(19.1%)	<0.001
Shock^‡^	59(3.5%)	16(1.3%)	19(6.6%)	24(10.4%)	<0.001
APACHE II^†^	15(12, 19)	14(11, 17)	17(14, 20)	19(15, 23)	<0.001
SOFA^†^	3(2, 5)	2(2, 4)	4(3, 5)	5(4, 6)	<0.001
GCS (ICU day 1)^†^	10(8, 10)	10(9, 10)	10(7, 10)	7(5, 10)	<0.001
ICU readmission^‡^	168(10.0%)	70(5.9%)	75(25.9%)	23(10.0%)	<0.001
ICU LOS (day)^†^	6(3, 13)	4(2, 7)	15(9, 24)	16(10, 26)	<0.001
Hospital LOS (day)^†^	21(15, 31)	19(14, 26)	30(23, 41)	29(22, 42)	<0.001
GOS^†^	4(3, 5)	5(4, 5)	4(3, 4)	3(3, 3)	<0.001
Death^‡^	111(6.6%)	71(6.0%)	21(7.2%)	19(9.4%)	0.176

^†^Data were expressed as median (IQR). ^‡^Data were expressed as frequency (%). CNS, central nervous system; AKI, acute Kidney Injury; APACHE, acute physiology and chronic health evaluation; SOFA, sequential organ failure assessment; GCS, Glasgow coma scale; ICU, intensive care unit; LOS, length of stay; GOS, Glasgow outcome scale.

Elective surgeries predominated (n = 1,413, 84.0% [95% CI 82.1–85.8]) versus emergency cases; infratentorial lesions were more frequent than supratentorial (n = 885, 52.6% [95% CI 50.2–55.0]) (Table 2). The primary surgical indications were intracranial tumors (n = 1,149, 68.3% [95% CI 66.0–70.5]), cerebrovascular diseases (n = 312, 18.5% [95% CI 16.7–20.5]), and traumatic brain injuries (n = 151, 9.0% [95% CI 7.7–10.5]), with a median operative duration of 4.9 hours (IQR 3.4–6.5). Compared to patients with infratentorial lesions, those with supratentorial lesions were older (52 vs 48 years, Z = −5.280, p < 0.001), more likely to be male (57.1% vs 48.4%, OR=1.424, 95% CI 1.174–1.726, p < 0.001), more frequently required emergency surgery (31.0% vs 2.6%, OR=16.801, 95% CI 10.815–26.098, p < 0.001), and had a higher prevalence of comorbidities, including hypertension (28.6% vs 21.6%, OR=1.453, 95% CI 1.164–1.814, p = 0.001), diabetes mellitus (11.0% vs 7.6%, OR=1.513, 95% CI 1.084–2.112, p = 0.014), tumors (5.9% vs 3.6%, OR=1.668, 95% CI 1.053–2.642, p = 0.028), and cerebrovascular diseases (6.8% vs 3.7%, OR=1.874, 95% CI 1.202–2.922, p = 0.005). Moreover, patients with supratentorial lesions more frequently underwent craniotomy for cerebrovascular diseases (25.9% vs 11.9%, OR=2.602, 95% CI 2.010–3.368, p < 0.001) or traumatic brain injuries (TBI) (18.3% vs 0.6%, OR=39.411, 95% CI 16.069–96.659, p < 0.001).

**Table 2:** Comparison of perioperative characteristics among patients undergoing direct tracheostomy versus those with successful or failed first extubation.

Variables	Total (n = 1683)	Success (n = 1190)	Failed (n = 290)	Tracheostomy (n = 230)	p value
Indications for craniotomy					
Tumor^‡^	312(18.5%)	222(18.7%)	48(16.6%)	42(18.3%)	0.026
Cerebrovascular disease^‡^	151(9.0%)	88(7.4%)	28(9.7%)	35(15.2%)	0.499
Trauma^‡^	71(4.2%)	57(4.8%)	10(3.4%)	4(1.7%)	<0.001
Other^‡^	1149(68. 3%)	823(69. 2%)	204(70.3%)	122(53.0%)	0.140
Surgical site					0.014
Supratentorial^‡^	798(47.4%)	543(45.63%)	140(48.3%)	115(50.0%)	
Infratentorial^‡^	885(52.6%)	647(54.4%)	150(51.7%)	88(38.3%)	
Duration of operation^†^	4.9(3.4, 6.5)	5(3.5, 6.7)	4.6(3.4, 6.5)	4.3(3.0, 6.2)	0.591
Admission category					<0.001
Elective surgery^‡^	1413(84.0%)	1016(85.4%)	249(85.9%)	148(64.3%)	
Emergency surgery^‡^	270(16.0%)	174(14.6%)	41(14.1%)	55(23.9%)	

^†^Data were expressed as median (IQR). ^‡^Data were expressed as frequency (%).

### 3.2. Extubation outcomes

**Fig 1:**
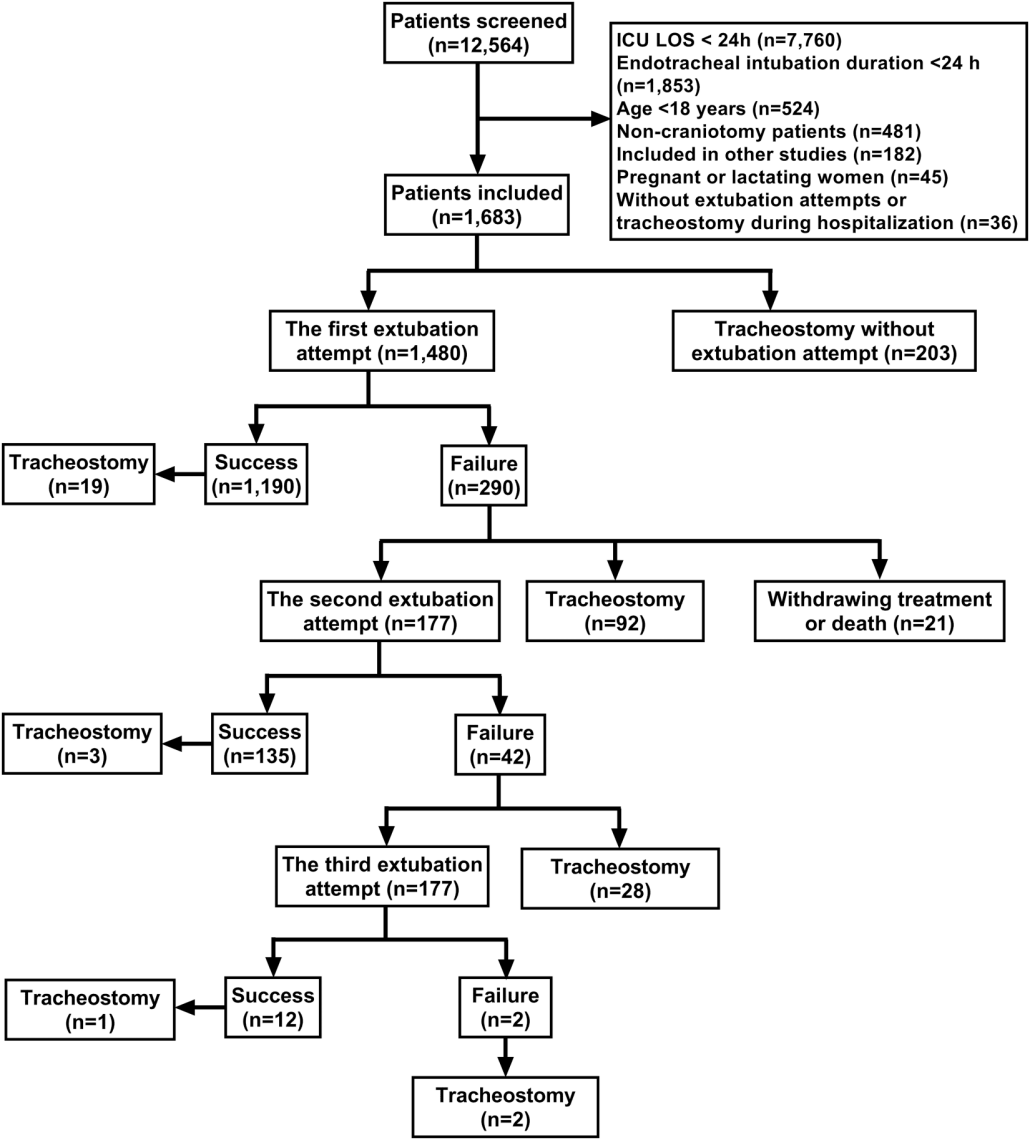
Patient screening and extubation outcomes flowchart. ICU, intensive care unit; LOS, Length of stay.

**Fig 2:**
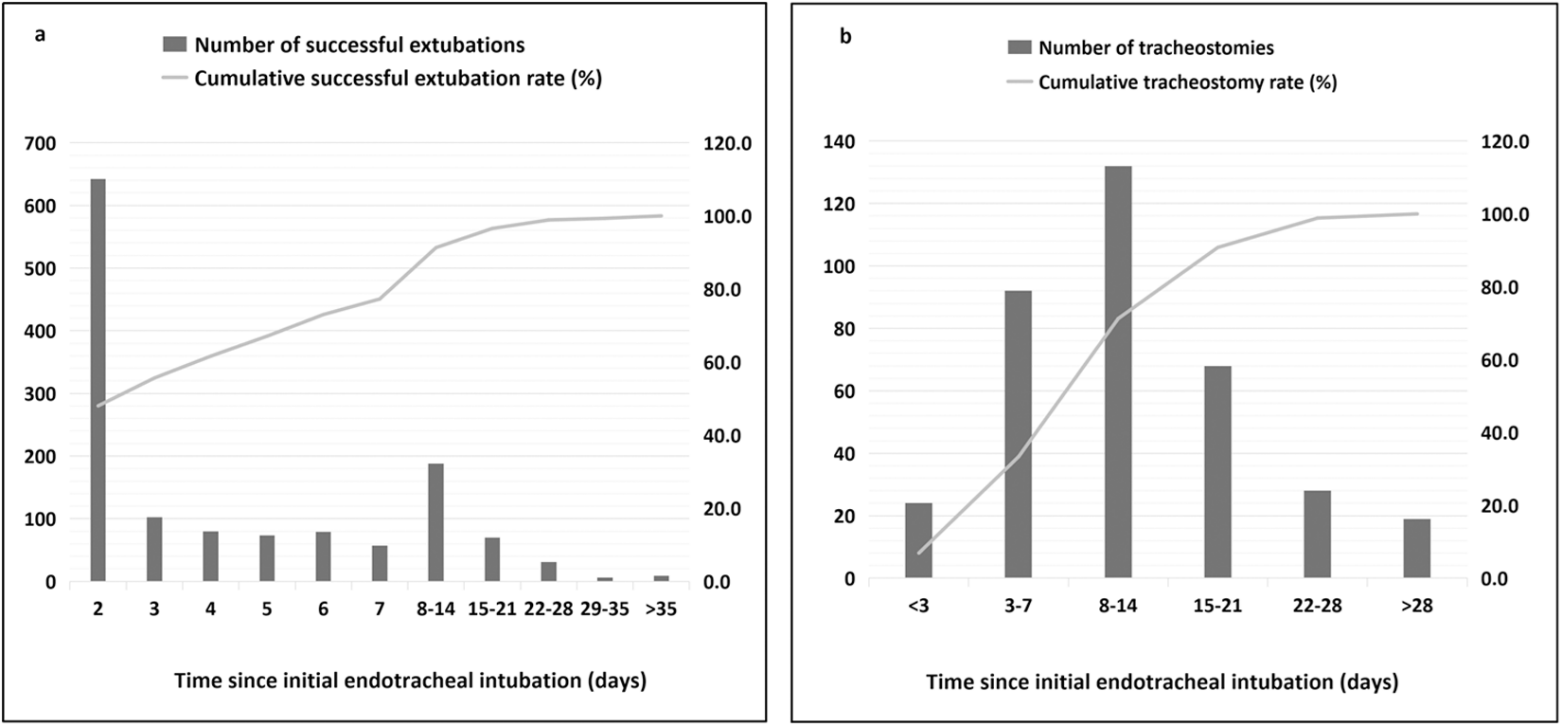
Time to successful extubation (a) and tracheostomy (b) in critically ill post-craniotomy patients.

Of 1,683 patients, 203 received direct tracheostomy and 1,480 underwent extubation attempts. First-attempt success rate reached 70.7% (1,190/1,683), leaving 290 failures (Fig 1). Subsequent attempts yielded 46.6% success (135/290) on second try and 28.6% (12/42) on third. Following successful first extubation, 19 patients required tracheostomy and 71 died during hospitalization. After successful second extubation, 3 patients underwent tracheostomy beyond 72 hours and 0 died during hospitalization. Among third-attempt successes, 1 patient received tracheostomy after 72 hours and 1 died during hospitalization. Among patients with initial extubation failure, 135 succeeded in the second attempt (3 subsequently underwent tracheostomy with 0 deaths), while 12 succeeded in the third attempt (1 received tracheostomy and 1 required reintubation with subsequent death). Ultimately, 142 patients with initial extubation failure achieved successful extubation and survived to discharge. A total of 1,244 patients were discharged without an artificial airway, representing an overall extubation success rate of 77.7%; 348 (20.7%) patients received tracheostomies during hospitalization; 27 (1.6%) patients either died or were discharged with endotracheal tubes. The most common cause of reintubation was refractory hypoxemia, followed by respiratory distress, inadequate airway secretion clearance, and neurological deterioration (Table 3). Median intubation durations were 2, 9, and 10 days for first-attempt success, failure, and direct tracheostomy groups respectively. Successful extubations occurred within 3 postoperative days in 55.6% of cases, within 7 days in 77.3%, and within 14 days in 91.3% (Fig 2).

**Table 3:** Reasons for reintubation among patients with extubation failure.

Reasons for reintubation	First reintubation (n = 290)^†^	Second reintubation (n = 42)^†^	Third reintubation (n = 2)^†^
Refractory hypoxemia	147 (50.7%)	23 (54.8%)	2 (100.0%)
Excessive work of breathing	104 (35.9%)	18 (42.9%)	1 (50.5%)
Difficulty managing secretions	84 (29.0%)	13 (31.0%)	0
Neurological deterioration	53 (18.3%)	4 (9.5%)	0
Respiratory acidosis	16 (5.5%)	2 (4.8%)	0
Laryngeal edema	8 (2.8%)	4 (9.5%)	0
Hemodynamic instability	8 (2.8%)	0	0
Severe agitation	5 (1.7%)	0	0
Cardiac arrest	1 (0.3%)	1 (2.4%)	0

^†^The total number of reasons for extubation failure exceeded the number of patients, as multiple factors might contribute to extubation failure in a single patient.

Comparative analysis showed reduced likelihood of first-attempt extubation success in older patients (per-year OR 0.980, 95%CI 0.973–0.988, p < 0.001), those with hypertension (OR 0.449, 95%CI 0.356–0.567, p < 0.001) or diabetes mellitus (OR 0.589, 95% CI 0.419–0.828, p = 0.002), patients with higher APACHE II (per-point OR 0.875, 95% CI 0.856–0.894, p < 0.001) and SOFA scores (per-point OR 0.729, 95% CI 0.693–0.767, p < 0.001), TBI patients (OR 0.545, 95% CI 0.387–0.767, p < 0.001), emergency surgery cases (OR 0.708, 95% CI 0.538–0.932, p = 0.014), and individuals with supratentorial lesions (OR 0.783, 95% CI 0.635–0.967, p = 0.023), while higher admission GCS scores predicted greater success rates (per-point OR 1.252, 95% CI 1.204–1.302, p = 0.014).

**Fig 3:**
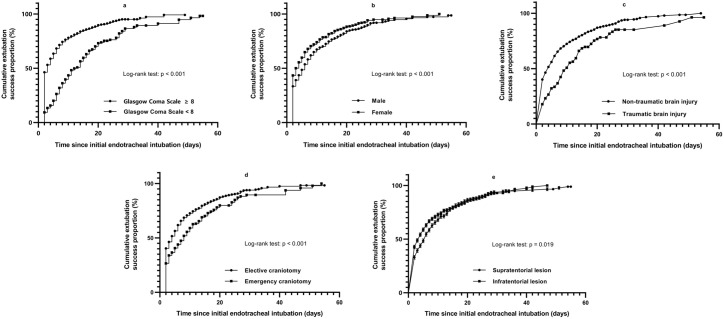
Kaplan-meier analysis of extubation success stratified by Glasgow coma scale, sex, surgical indications, admission types, and lesion locations.

Intergroup comparisons demonstrated significantly lower extubation success rates at discharge for male patients compared to females (75.2% vs 80.5%, OR=0.737, 95% CI 0.584–0.929, p = 0.010), emergency surgeries versus elective surgeries (69.6% vs 79.3%, OR=0.600, 95% CI 0.449–0.801, p < 0.001), supratentorial lesion patients relative to infratentorial cases (75.6% vs 79.7%, OR=0.790, 95% CI 0.627–0.994, p = 0.046), and TBI patients compared to non-TBI cases (64.2% vs 79.0%, OR=0.476, 95% CI 0.334–0.679, p < 0.001). Kaplan-Meier analysis (Fig 3) confirmed these findings and additionally revealed poorer extubation outcomes in patients with GCS < 8 (56.1% vs 83.9%, OR=0.254, 95% CI 0.197–0.328, p < 0.001).

### 3.3. Clinical outcomes

Postoperative complications following craniotomy included infections in 49.6% of cases (predominantly pneumonia and central nervous system infections), sepsis in 23.2%, AKI in 11.1%, and shock in 3.5%. Patients with successful first extubation attempts exhibited significantly reduced complication rates across all categories compared to both extubation failure and tracheostomy cohorts. Patients with successful first extubation demonstrated superior clinical outcomes across multiple parameters compared to both extubation-failure and tracheostomy cohorts. Specifically, the successful extubation group had significantly shorter median duration of mechanical ventilation (2 days, IQR 2−6 vs. 10 days, IQR 6−16 in failure group and 9 days, IQR 6−13 in tracheostomy group; p < 0.001), reduced median hospitalization costs (76,366 CNY, IQR 57,805−106,883 vs. 147,170 CNY, IQR 105,990−193,770 in failure group and 149,242 CNY, IQR 120,971−192,699 in tracheostomy group; p < 0.001), and better neurological outcomes as assessed by discharge GOS scores. The overall cohort had median ICU and hospital stays of 6 and 21 days respectively. Patients with successful first extubation required significantly shorter hospitalization and ICU LOS than both comparison groups (p < 0.001), while no significant difference was observed between failure and tracheostomy patients.

Compared with late tracheostomy patients, early tracheostomy showed no significant differences in complications including pneumonia, CNS and surgical site infections, sepsis, AKI or shock, nor in hospital mortality or discharge GOS scores, but demonstrated shorter ICU and hospital LOS, lower ICU readmission rates, and reduced hospitalization costs.

The overall hospital mortality rate was 6.6%. Deceased patients exhibited significantly older age (median 54 vs 49 years; p = 0.001) and higher comorbidity burdens, including hypertension (34.2% vs 24.2%; p = 0.019), diabetes mellitus (16.2% vs 8.7%; p = 0.008), cerebrovascular disease (9.9% vs 4.8%; p = 0.020), and coronary artery disease (7.2% vs 2.7%; p = 0.014). These patients also demonstrated greater incidence of critical complications, including sepsis (49.5% vs 21.4%; p < 0.001), acute kidney injury (48.6% vs 8.5%; p < 0.001) and shock (25.2% vs 2.0%; p < 0.001). Clinically, the deceased group showed more severe physiological derangements, evidenced by higher APACHE II (23 vs 15; p < 0.001) and SOFA scores (6 vs 3; p < 0.001), along with lower initial GCS scores (5 vs 10; p < 0.001). They had prolonged mechanical ventilation (7 vs 4 days; p < 0.001) and ICU LOS (9 vs 6 days; p < 0.001), yet paradoxically shorter total hospitalization (16 vs 21 days; p < 0.001). Notably, mortality rates differed significantly by anatomical location (supratentorial: 9.6% vs infratentorial: 3.8%; p < 0.001) and etiology (traumatic: 14.5% vs non-traumatic: 6.3%; p < 0.001). Initial extubation success status showed no significant association with mortality outcomes. The most common cause of death was cerebral herniation secondary to intracranial complications (n = 78), followed by septic shock (n = 13), severe pneumonia (n = 8), and cardiogenic shock (n = 7).

### 3.4. Predictors of successful first extubation

Multivariate regression analysis identified female sex, absence of hypertension, infratentorial lesions, lower SOFA scores, and reduced APACHE II scores as independent predictors of successful first extubation (Table 4).

**Table 4:** Logistic regression analysis of factors influencing initial extubation success.

Variables	OR	95% CI	p value
Male	0.680	0.531, 0.871	0.002
Hypertension	0.637	0.489, 0.829	0.001
Supratentorial lesion	1.315	1.016, 1.701	0.037
SOFA	0.844	0.791, 0.900	<0.001
APACHE II	0.930	0.902, 0.958	<0.001

OR, odds ratio; CI, confidence interval; SOFA, sequential organ failure assessment; APACHE, acute physiology and chronic health evaluation.

## 4. Discussion

This study demonstrated a first-attempt extubation success rate of 70.7% among critically ill post-craniotomy patients, with progressively declining success rates in subsequent attempts (46.6% for second attempt and 28.6% for third attempt). The multivariate analysis revealed consistent predictive factors for first-attempt success, including female sex, normotensive status, infratentorial lesion location, and lower SOFA and APACHE II scores.

This investigation revealed a 19.6% first-attempt extubation failure rate among post-craniotomy patients, aligning with published neurocritical care benchmarks (10–22.6%) [4, 6, 16, 27–29] but remaining numerically lower than McCredie et al.‘s reported 21% [4]. The observed discrepancy may stem from fundamental cohort differences. While their study population consisted predominantly of TBI cases (including only 6.3% who underwent craniotomy), our investigation specifically focused on craniotomy patients, among whom 68.3% presented with intracranial neoplasms. Our results revealed significantly lower first-extubation success rates in TBI patients compared with non-TBI patients. This marked difference in patient characteristics likely accounted for the observed variations in extubation outcomes. Notably, their cohort exhibited lower GCS at ICU admission, suggesting greater disease severity, which may partially explain their higher extubation failure rates. The failure rate in our study was significantly higher than the 11.3% reported by Angriman et al. Their lower mortality rate suggests that the enrolled patients likely had less severe conditions, which may account for the lower extubation failure rate compared to our study [30]. Variations in defining extubation failure may contribute to divergent study outcomes. While most studies use 72-hour reintubation as the standard [4, 31], we similarly classified reintubation within 72 hours as failure to maintain consistency with prior research. However, Godet et al. utilized a 48-hour threshold [32], while Cinotti et al. employed a 5-day cutoff [33]. We suggest that a 48-hour cutoff may underestimate extubation failure rates, while a 5-day threshold would introduce significant confounding from factors like pulmonary infections, complicating both outcome assessment and risk factors identification. Current extubation failure criteria lack standardization, and influencing factors may vary individually, for instance, delayed neurological complications (intracranial hypertension, hydrocephalus, or cerebral herniation) may manifest during subacute recovery (days-to-weeks post-injury) [34] – making it challenging to establish universally applicable diagnostic criteria.

Our findings demonstrate that a clinically significant subset of patients (142/290) with initial extubation failure ultimately achieved successful airway liberation. Although delayed tracheostomy implementation was associated with prolonged durations of both mechanical ventilation and hospitalization [35–37], avoiding the procedure could prevent procedure-related complications, including tracheostomy site infections, hemorrhage, and tracheoesophageal fistula formation [38]. These data support consideration of a carefully monitored observation period for selected neurosurgical patients following initial extubation failure, while emphasizing the need for individualized decision-making that balances the risks of delayed intervention against the potential benefits of avoiding permanent airway alterations.

The results of this study showed comparable mortality rates across initial extubation success, extubation failure and direct tracheostomy groups. Analysis revealed that brain herniation resulting from various intracranial complications was the most common cause of death across all groups, including those who were initially extubated successfully, likely explaining the similar mortality rates. These findings suggest that even after successful initial extubation, critically ill post-craniotomy patients remain at risk of reintubation or death due to intracranial complications, highlighting the need for vigilant monitoring to detect and mitigate such complications. The ICU and hospital LOS in the tracheostomy group were significantly longer than those in the initial extubation success group but showed no difference compared to the initial extubation failure group. This finding was highly consistent with McCredie et al.‘s report that direct tracheostomy failed to improve mortality while correlating with prolonged ICU and hospital stays [4]. Notably, our direct tracheostomy group showed significantly higher rates of pulmonary infections, sepsis, and shock compared to initial extubation success patients. Furthermore, their lower GCS, higher APACHE II and SOFA scores indicated greater illness severity, likely contributing to prolonged hospitalization.

The clinical utility of early tracheostomy in critically ill post-craniotomy patients remains unclear. Alali et al. found that while early tracheostomy failed to reduce mortality, it significantly decreased mechanical ventilation duration and shortened both ICU and hospital stays [39]. Our subgroup analyses corroborated these findings, demonstrating reduced ICU/hospital stays and lower hospitalization costs without mortality benefits or improved discharge GOS scores. These results align with a meta-analysis showing that early tracheostomy in acute brain injury patients reduced ventilation time and improved long-term survival, though without short-term mortality benefit [40]. However, our study’s lack of long-term follow-up data limited evaluation of functional recovery outcomes. Future large-scale prospective studies are needed to comprehensively assess early tracheostomy’s impact on both acute and long-term outcomes in this patient population.

Multivariate logistic regression analysis incorporating demographic, comorbidity, surgical, and illness severity variables identified five independent predictors of successful first extubation, which were female sex, absence of hypertension, infratentorial lesion, lower SOFA and lower APACHE II score. Given the paucity of studies examining predictors of extubation outcomes in critically ill post-craniotomy patients, direct comparison of our findings with existing literature was challenging.

While previous studies suggested low GCS scores as a predictor of extubation failure [6], our findings revealed no significant association between GCS and extubation outcomes. First, inter-rater variability may exist in GCS scoring; moreover, GCS scores tend to fluctuate over time, making them unreliable as a gold standard for predicting extubation outcomes. Notably, both SOFA and APACHE II scores—which incorporate GCS as a component—emerged as independent predictors. This paradox underscores that extubation success in post-craniotomy critically ill patients depends not solely on neurological status (as reflected by GCS), but rather on multisystem organ function, as captured by these composite severity scores.

Contrary to neuroanatomical expectations, patients with infratentorial lesions demonstrated significantly higher extubation success rates compared to supratentorial cases, despite the latter’s theoretically lesser impact on brainstem-mediated airway protective reflexes (cough and swallowing functions). Subsequent analysis demonstrated that patients with supratentorial lesions had significantly higher prevalence of hypertension, diabetes mellitus, emergency surgeries, and traumatic etiologies compared to infratentorial cases. The complex interactions among these confounding factors may account for the observed versus expected outcome discrepancies. Similarly, the higher first-attempt success rate in female patients might be associated with lower frequencies of diabetes (6.9% vs 11.3%, p = 0.002), hypertension (22.2% vs 27.4%, p = 0.013), emergency procedures (13.6% vs 18.2%, p = 0.011), traumatic brain injuries (6.9% vs 10.9%, p = 0.004), and supratentorial lesions (48.4% vs 57.2%, p < 0.001). These findings strongly suggest that comprehensive extubation decision-making in neurosurgical patients should incorporate multivariate assessment of gender, comorbidities, and surgical characteristics alongside traditional neurological evaluations to enhance predictive accuracy.

Our study evaluated extubation outcomes and their influencing factors in post-craniotomy ICU patients. However, several methodological limitations merit consideration in interpreting these findings. First, as a single-center study conducted at a specialized neurological care facility with considerable expertise in managing heterogeneous neurocritical populations, our findings may have limited immediate generalizability to other clinical settings, although our high patient volume and diverse case exposure provide valuable preliminary insights. Second, the retrospective study design inherently restricted the systematic assessment of all potential risk factors for extubation outcomes. Specifically, we were unable to evaluate clinically significant parameters such as diaphragmatic dysfunction [41] (commonly seen in neurocritical patients due to primary neurological insults and ICU-acquired weakness) and respiratory secretion burden (including sputum volume [42, 43] and suction frequency [44]), both known to impact extubation success. Additionally, postoperative complications were identified through medical record review, diagnostic accuracy might be affected by documentation completeness. Furthermore, the retrospective design precluded control for sedative use, ventilator settings, or ICU management practices, all of which may potentially influence weaning and extubation outcomes. Third, the observational nature prevents causal inferences regarding the bidirectional relationship between complications and extubation failure. For example, while respiratory infections may precipitate extubation failure, prolonged intubation itself increases ventilator-associated pneumonia risk. Fourth, while institutional protocol mandated multidisciplinary evaluation (intensivist and neurosurgical teams) for all extubation decisions, unquantified confounders including socioeconomic factors and family preferences may have introduced allocation bias. Additionally, individual patient variations meant that some cases meeting extubation criteria might still have been withheld based on comprehensive clinical judgment. Furthermore, the 4-year study period may introduce temporal heterogeneity due to evolving clinical protocols, particularly regarding extubation practices, which, while remaining largely unchanged over the past decade, could still potentially impact study results. Finally, the extubation-influencing factors identified in this study require further external validation. These limitations highlight the need for prospective studies incorporating protocolized weaning parameters, serial physiological measurements, and standardized decision documentation to better identify true predictors of extubation success in this population.

## 5. Conclusions

This study demonstrates extubation success rates of 70.7%, 46.6%, and 28.6% for first, second, and third attempts respectively in post-craniotomy critically ill patients. Multivariate regression analysis identified female sex, absence of hypertension, infratentorial lesions, lower SOFA scores, and lower APACHE II scores as independent predictors of first-attempt success. As one of the few systematic investigations in this area, these findings might offer valuable insights for clinical extubation decisions and may inform future prospective studies, particularly regarding extubation outcome predictors and optimal tracheostomy timing.
