# Correction: Extubation outcomes in critically ill post-craniotomy patients: A retrospective cohort study

**DOI:** 10.1371/journal.pone.0343465

**Published:** 2026-02-24

**Authors:** Jianfang Zhou, Xu-Ying Luo, Guangzhi Shi, Hong-Liang Li, Guang-Qiang Chen

## Notice of Republication

This article [1] was republished on October 21, 2025, to address an issue identified post-publication. An updated version of S1 File is provided with this notice. Please download this article again to view the correct version.

The article’s Data Availability statement is updated to: All relevant data are within the manuscript and its Supporting Information file.

In addition to the concerns above, the corresponding author contacted PLOS about errors in Fig 1, and Tables 1–2;

- In Fig 1, the number of cases for the third extubation attempt is incorrectly duplicated from the second attempt; the correct value is 14, not 177. The correct Fig 1 is provided here.
- In Tables 1 and 2, in the fifth column, the number of tracheostomy cases are erroneously recorded as 230, while the correct value is 203. These errors were carried over into the percentage calculations within the same columns, however, the p-values are correct. The correct Tables 1 and 2 with updated tracheostomy case percentages are provided here.
- In Table 2, the case numbers for different craniotomy indications (rows 3–6) are misaligned with their corresponding column headings. Please see the correct Table 2 below.

**Table 1:** Demographic and outcome comparisons among direct tracheostomy patients and those with first extubation success or failure.

Variables	Total (n = 1683)	Success (n = 1190)	Failed (n = 290)	Tracheostomy (n = 203)	p value
Age (year)^†^	50(38, 59)	48(36, 57)	54(44, 63)	53(40, 60)	<0.001
Male^‡^	884(52.5%)	583(49.0%)	183(63.1%)	118(58.1%)	<0.001
Smoking^‡^	256(15.2%)	162(13.6%)	58(20.0%)	36(17.7%)	0.014
Alcoholism^‡^	129(7.7%)	80(6.7%)	31(10.7%)	18(8.9%)	0.059
Comorbidities					
Hypertension^‡^	419(24.9%)	241(20.3%)	117(40.3%)	61(30.0%)	<0.001
Diabetes^‡^	155(9.2%)	93(7.8%)	32(11.0%)	30(14.8%)	0.003
Tumor^‡^	79(4.7%)	50(4.2%)	22(7.6%)	7(3.4%)	0.034
Cerebrovascular disease^‡^	87(5.2%)	57(4.8%)	17(5.9%)	13(6.4%)	0.531
Coronary heart disease^‡^	50(3.0%)	30(2.5%)	12(4.1%)	8(3.9%)	0.238
Chronic lung disease^‡^	9(0.5%)	6(0.5%)	2(0.7%)	1(0.5%)	0.929
Pneumonia^‡^	540(32.1%)	214(18.0%)	190(65.5%)	136(67.0%)	<0.001
CNS infection^‡^	387(23.0%)	257(21.6%)	78(26. 9%)	52(25.6%)	0.101
Surgical site infection^‡^	39(2.3%)	19(1.6%)	10(3.4%)	10(4.9%)	0.005
Sepsis^‡^	391(23.2%)	151(12.7%)	142(49.0%)	98(48.3%)	<0.001
AKI^‡^	187(11.1%)	96(8.1%)	47(16.2%)	44(21.7%)	<0.001
Shock^‡^	59(3.5%)	16(1.3%)	19(6.6%)	24(11.8%)	<0.001
APACHE II^†^	15(12, 19)	14(11, 17)	17(14, 20)	19(15, 23)	<0.001
SOFA^†^	3(2, 5)	2(2, 4)	4(3, 5)	5(4, 6)	<0.001
GCS (ICU day 1)^†^	10(8, 10)	10(9, 10)	10(7, 10)	7(5, 10)	<0.001
ICU readmission^‡^	168(10.0%)	70(5.9%)	75(25.9%)	23(11.3%)	<0.001
ICU LOS (day)^†^	6(3, 13)	4(2, 7)	15(9, 24)	16(10, 26)	<0.001
Hospital LOS (day)^†^	21(15, 31)	19(14, 26)	30(23, 41)	29(22, 42)	<0.001
GOS^†^	4(3, 5)	5(4, 5)	4(3, 4)	3(3, 3)	<0.001
Death^‡^	111(6.6%)	71(6.0%)	21(7.2%)	19(9.4%)	0.176

^†^ Data were expressed as median (IQR). ‡ Data were expressed as frequency (%). CNS, central nervous system; AKI, acute Kidney Injury; APACHE, acute physiology and chronic health evaluation; SOFA, sequential organ failure assessment; GCS, Glasgow coma scale; ICU, intensive care unit; LOS, length of stay; GOS, Glasgow outcome scale.

**Table 2:** Comparison of perioperative characteristics among patients undergoing direct tracheostomy versus those with successful or failed first extubation.

Variables	Total (n = 1683)	Success (n = 1190)	Failed (n = 290)	Tracheostomy (n = 203)	p value
Indications for craniotomy					
Tumor^‡^	1149(68.3%)	823(69.2%)	204(70.3%)	122(60.1%)	0.026
Cerebrovascular disease^‡^	312(18.5%)	222(18.7%)	48(16.6%)	42(20.7%)	0.499
Trauma^‡^	151(9.0%)	88(7.4%)	28(9.7%)	35(17.2%)	<0.001
Other^‡^	71(4.2%)	57(4.8%)	10(3.4%)	4(2.0%)	0.140
Surgical site					0.014
Supratentorial^‡^	798(47.4%)	543(45.6%)	140(48.3%)	115(56.7%)	
Infratentorial^‡^	885(52.6%)	647(54.4%)	150(51.7%)	88(43.3%)	
Duration of operation(hour)^†^	4.9(3.4, 6.5)	5.0(3.5, 6.7)	4.6(3.4, 6.5)	4.3(3.0, 6.2)	0.591
Admission category					<0.001
Elective surgery^‡^	1413(84.0%)	1016(85.4%)	249(85.9%)	148(72.9%)	
Emergency surgery^‡^	270(16.0%)	174(14.6%)	41(14.1%)	55(27.1%)	

^†^ Data were expressed as median (IQR). ^‡^ Data were expressed as frequency (%).

After follow-up with the corresponding author and consultation with a member of the Editorial Board, the *PLOS One* Editors determined that the above errors do not affect the main conclusions of [1].

**Fig 1:**
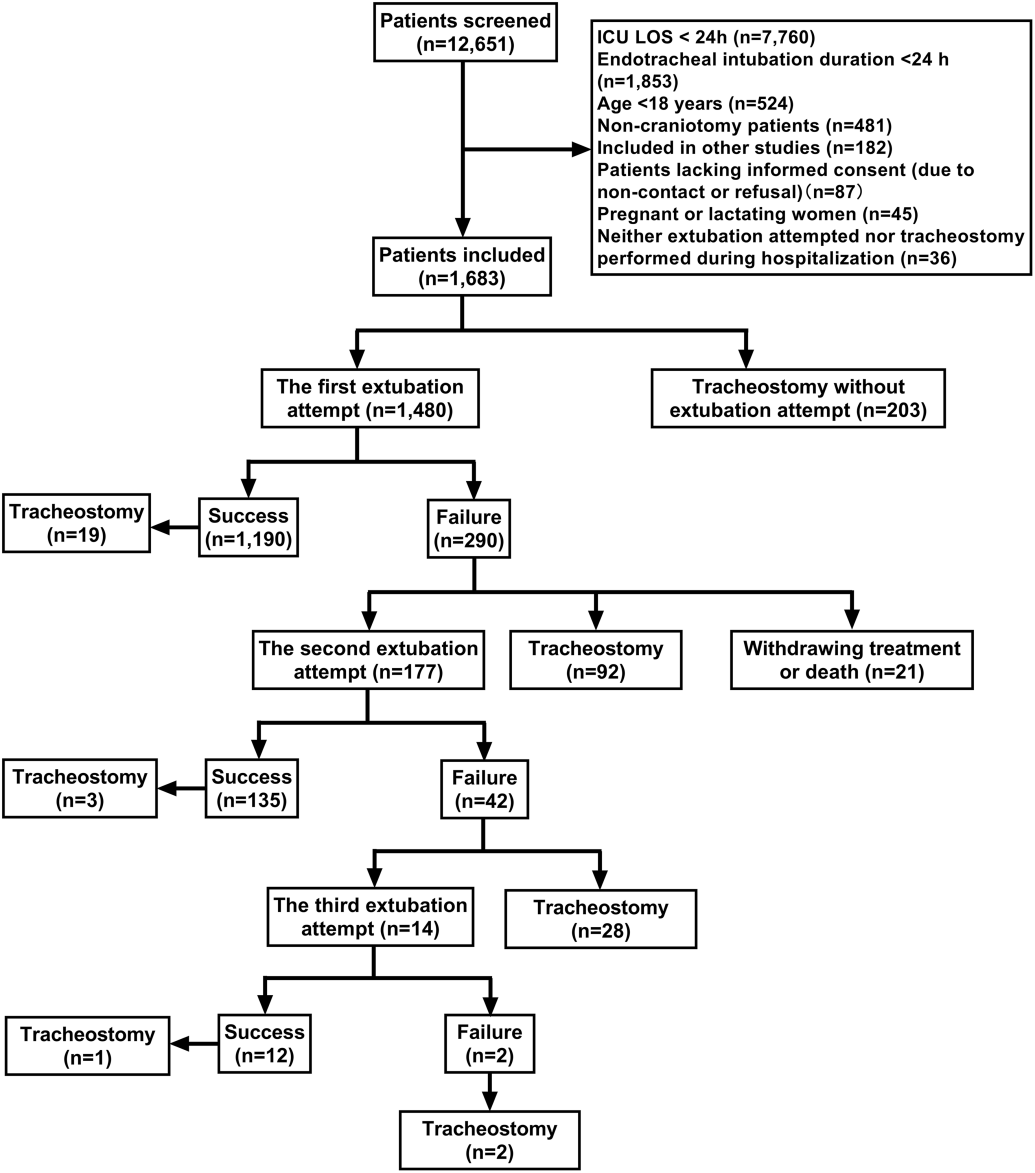
Patient screening and extubation outcomes flowchart. ICU, intensive care unit; LOS, Length of stay.

## Supporting information

S1 File Raw data. (XLSX)
